# Mesoamerican nephropathy: A silent epidemic at the nexus of climate, labor, and health

**DOI:** 10.3934/publichealth.2026035

**Published:** 2026-05-28

**Authors:** J. Luis Espinoza, Leyla Abdalah-Perez

**Affiliations:** 1 Faculty of Health Sciences, Kanazawa University, Kanazawa, Japan; 2 Department of Medicine, Baptist Hospital of Nicaragua; 3 Renal Unit, Baptist Hospital of Nicaragua

**Keywords:** Mesoamerican nephropathy, environmental health, occupational kidney disease, neglected diseases, environmental toxins

## Abstract

Mesoamerican nephropathy (MeN) has emerged as a critical yet often overlooked occupational and environmental health crisis. Primarily affecting young, otherwise healthy agricultural workers in Central America, this disease leads to rapid progression to kidney failure without traditional causes like diabetes or hypertension. While the central drivers are recurrent heat stress and chronic dehydration, emerging research reveals a multifactorial pathogenesis. This includes synergistic nephrotoxic insults from agrochemicals, heavy metals, chronic endotoxin exposure, and mycotoxins (e.g., ochratoxin A). Morphologic studies point to shared pathways of tubular injury, characterized by mitochondrial dysfunction and lysosomal abnormalities. Furthermore, gut–kidney crosstalk and genetic susceptibility, particularly among individuals with Native American ancestry, may amplify renal inflammation and injury. Although targeted interventions, such as enhanced hydration, rest, and access to shade, show promise, their efficacy in halting disease progression remains limited. As global temperatures rise, similar disease patterns are now being reported among outdoor laborers in other hot regions, signaling a broader climate-linked public health threat. Addressing MeN demands a concerted, multidisciplinary effort encompassing rigorous pathogenesis research, enforceable occupational protections, and global recognition of heat-associated kidney disease as a growing epidemic. This perspective synthesizes recent insights into MeN and calls for urgent, actionable measures to confront this silent crisis.

## 1. Introduction

Mesoamerican nephropathy (MeN), also referred to as chronic kidney disease (CKD) of nontraditional origin (CKDnt), is a rapidly progressive tubulointerstitial nephropathy affecting predominantly young agricultural workers without traditional risk factors such as diabetes or hypertension. Community-based studies in Nicaragua and El Salvador report CKD prevalence exceeding 15%–20% among working-age men in affected regions [1].

Imagine a 29-year-old man, robust and in the prime of his life, collapsing under the relentless midday sun in a vast sugarcane field within the Pacific lowlands of Nicaragua. He has no prior medical history of diabetes, hypertension, or other chronic illnesses. Yet, upon evaluation, he is found to have advanced kidney failure, with creatinine levels soaring and kidneys shrunken on ultrasound. Despite efforts to manage his symptoms, without affordable access to dialysis or a transplant, he succumbs within a year. This tragic narrative is not an isolated incident but a stark representation of MeN, a silent epidemic claiming thousands of lives annually in Central America.

The epidemic was first systematically recognized in the early 2000s when clinicians in El Salvador and Nicaragua observed unusually high rates of kidney failure among young male agricultural workers. Retrospective mortality analyses later revealed rising CKD-related deaths beginning in the 1990s, particularly in Pacific coastal regions. Early hypotheses focused on agrochemical toxicity, but subsequent epidemiologic investigations increasingly highlighted recurrent occupational heat stress and dehydration as central drivers. The evolution of the MeN hypothesis—from toxic exposure to a multifactorial model incorporating climate, labor, environmental toxins, gut permeability, and genetic susceptibility—reflects two decades of interdisciplinary research across nephrology, occupational health, and environmental epidemiology [2],[3]. Prevalence is highest in the Pacific coastal regions of El Salvador and Nicaragua, where community-based studies report CKD rates exceeding 20% among working-age men, but cases extend to Costa Rica, Guatemala, Honduras, Panama, and southern Mexico [4].

The human toll is immense: Families are shattered by the loss of primary breadwinners, communities face labor shortages that perpetuate poverty cycles, and healthcare systems in low-resource settings are overwhelmed. Epidemiological surveys have estimated that over 50,000 individuals may have died from MeN-related end-stage kidney disease (ESRD) since the 1990s, though underreporting due to inadequate health infrastructure and diagnostic capabilities likely inflates this figure significantly. In El Salvador, for instance, MeN accounts for up to 70% of ESRD cases in certain hotspots, straining limited dialysis centers [5]. This crisis unfolds against a backdrop of socioeconomic vulnerability, where workers earn meager wages—often less than $10 per day—and lack basic protections like health insurance or regulated work hours [4]. As climate change drives rising temperatures and more frequent heatwaves, the geographic and demographic scope of MeN is expanding, raising alarms about a potential global surge in occupational kidney diseases.

This article aims to synthesize the current understanding of MeN's epidemiology, etiology, pathophysiology, and public health implications, while highlighting recent advances and gaps in knowledge. By drawing on multidisciplinary evidence from occupational health, epidemiology, and toxicology to genetics and climate science, we advocate for a comprehensive, equity-focused response to this underrecognized threat.

## 2. Epidemiology: Mapping the burden and patterns

**Figure 1.**
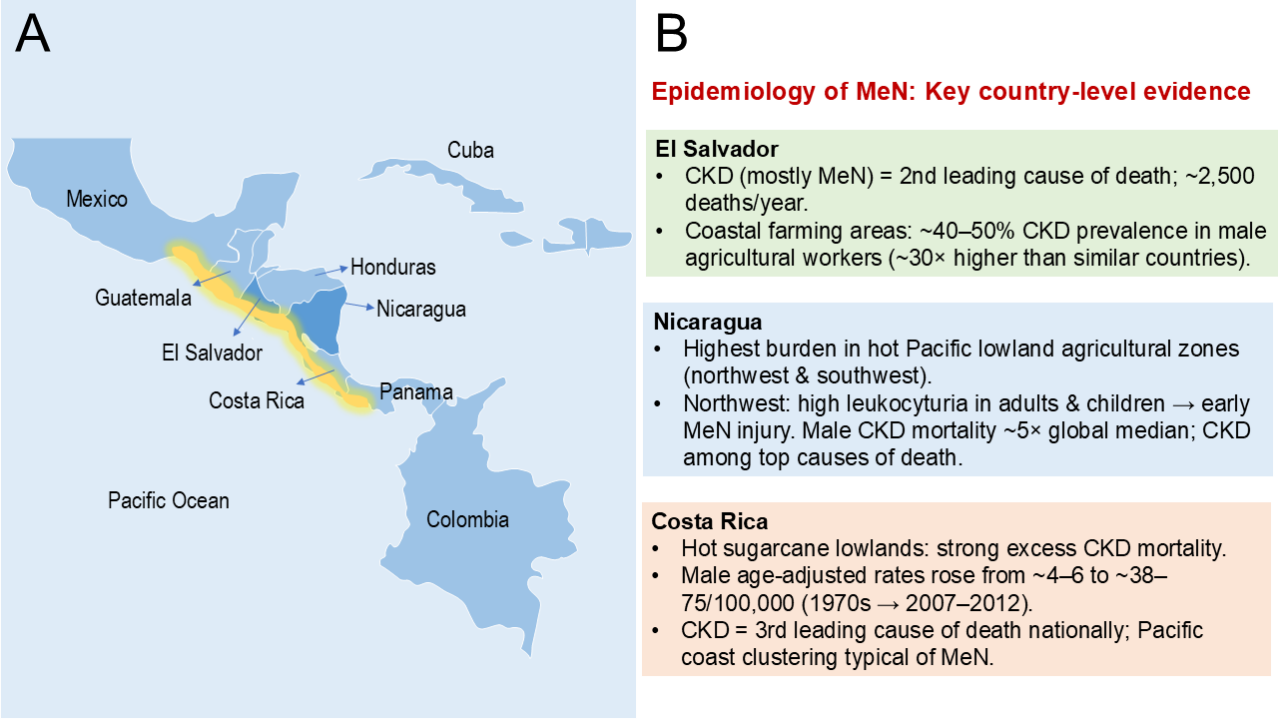
(A) Mesoamerican nephropathy (MeN) prevalence in Pacific regions of various countries of the Mesoamerican region, with El Salvador and Nicaragua having the highest prevalence, followed by Costa Rica, Guatemala, Panama, and some areas in the South of Mexico. (B) Epidemiology of MeN: Key country-level evidence from El Salvador, Nicaragua, and Costa Rica. CKD, chronic kidney disease.

MeN's epidemiology reveals a striking pattern of geographic clustering and occupational specificity. Hotspots are concentrated in low-altitude (<500 meters) Pacific coastal plains with average temperatures exceeding 30°C (86°F) and high humidity, conditions that exacerbate heat stress during peak agricultural seasons [6],[7]. In Nicaragua's León and Chinandega departments, cohort studies have shown a prevalence of reduced estimated glomerular filtration rates (eGFRs) of < 60 mL/min/1.73 m²) in up to 18% of male agricultural workers, compared with less than 5% in nonagricultural controls [8]. Similarly, in El Salvador's Bajo Lempa region, autopsy data indicate that CKD is the second leading cause of death among men aged 20–59 [6], while in Costa Rica, age-adjusted CKD mortality among men displays strong geographic clustering along the Pacific coast, consistent with MeN. In this region, mortality rates increased by approximately 9.5- to 12.5-fold, rising from 4–6 per 100,000 in the early 1970s to 38–75 per 100,000 in 2007–2012, far exceeding rates in the rest of the country [9] (Figure 1).

Demographically, MeN exhibits a strong male predominance (a male-to-female ratio of 3–5:1), attributed to gender-based labor divisions, where men perform the most strenuous fieldwork. However, emerging reports suggest increasing cases among women in domestic roles exposed to contaminated water or secondary heat stress. Age at onset is notably young, with the median diagnosis around 30 years, and progression to ESRD occurs within 5–10 years, far faster than typical CKD [7],[10].

Surveillance challenges compound the issue. Many affected areas lack routine kidney function testing, and death certificates often misattribute fatalities to “renal failure” without specifying the etiology. The Pan American Health Organization (PAHO) estimates that the true incidence may be 2–3 times higher than reported. Longitudinal studies, like those from the Consortium on the Epidemic of Nephropathy in Central America and Mexico (CENCAM), have tracked over 1000 workers, revealing seasonal spikes in acute kidney injury (AKI) during harvest periods, which correlate with long-term eGFR decline.

**Figure 2.**
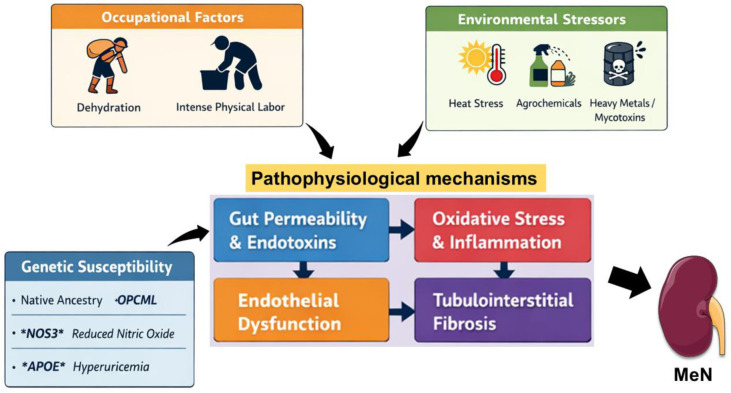
Multifactorial etiological model of Mesoamerican Nephropathy (MeN). This schematic illustrates the interplay of environmental, occupational, and biological factors contributing to MeN. Recurrent heat stress and dehydration lead to repeated subclinical acute kidney injury and cumulative tubular damage. Environmental exposures, including mycotoxins, agrochemicals, heavy metals, and endotoxin-contaminated water, promote oxidative stress and chronic inflammation, while impaired gut integrity facilitates systemic inflammatory responses via the gut–kidney axis. Genetic susceptibility, including variants in *OPCML, NOS3*, and *APOE*, may modulate vulnerability through effects on fluid balance, endothelial function, and oxidative stress. These mechanisms act synergistically to drive renal fibrosis and progressive kidney dysfunction.

Globally, parallels to MeN are evident in other tropical agrarian regions. In Sri Lanka's North Central Province, a similar entity termed Sri Lankan nephropathy or CKDu affects rice paddy farmers, with prevalence rates of 15%–20% [11]. In India, particularly in the Uddanam region, analogous clusters are linked to heat, agrochemicals, and water quality [12]. In the United States, agricultural workers in California's Central Valley and Florida's sugarcane fields show elevated CKD risks, with a 2021 study reporting threefold higher odds among those with high heat exposure [13]. These patterns underscore MeN as a harbinger of climate-amplified health disparities. Nevertheless, the etiology of MeN defies simplistic explanations, evolving from an initial focus on heat stress to a complex interplay of environmental, occupational, biological, and genetic factors (Figure 2). While no single cause has been definitively proven, convergent evidence supports a synergistic model where repeated insults culminate in irreversible renal damage.

## 3. Heat stress and dehydration: The core hypothesis

Central to MeN's pathogenesis is recurrent heat stress nephropathy [14]. Strenuous labor in hot, humid environments leads to core body temperature elevations, profuse sweating, and volume depletion. This triggers subclinical AKI episodes, characterized by elevated serum creatinine, urinary biomarkers like neutrophil gelatinase-associated lipocalin, and tubular injury. Over time, repeated AKI-to-CKD transitions promote interstitial fibrosis and tubular atrophy. Supporting evidence includes occupational cohort studies showing cross-shift eGFR declines during sugarcane harvests, with risks amplified by inadequate hydration [15]. Animal models in which rodents are repeatedly exposed to heat and dehydration develop renal oxidative stress, tubulointerstitial injury, and fibrosis, mirroring the lesions suspected in MeN [16]. Climate analyses show that MeN hotspots already experience large numbers of days each year above the recommended occupational heat thresholds and are projected to see substantial further increases in such days by mid‑century. Similar heat‑related kidney injury has been documented among agricultural workers in South Asia, and recent reviews warn that rising global temperatures may accelerate a worldwide epidemic of heat‑ and work‑related CKD in vulnerable labor populations [17]. However, heat alone does not explain MeN's regional specificity, as similar labor conditions exist elsewhere without comparable CKD burdens. This has prompted explorations of the cofactors.

## 4. Environmental toxins: Endotoxins, mycotoxins, and heavy metals

Contaminated drinking water is a prime suspect. In rural Mesoamerica, reliance on shallow wells and surface water exposes communities to Gram-negative bacteria, which release lipopolysaccharides (LPSs/endotoxins). LPS activates Toll-like receptor 4 (TLR4) in the renal cells, inducing inflammation via NF-κB pathways. Biomonitoring in Nicaraguan workers has detected elevated urinary LPS levels, and rodent studies show that LPS exacerbates heat-induced AKI [17].

Mycotoxins from fungal‑contaminated staples, particularly ochratoxin A (OTA) produced by *Aspergillus* and *Penicillium* species during humid storage, represent a plausible additional nephrotoxic exposure in MeN‑endemic regions. OTA is a well‑established tubulointerstitial nephrotoxin that causes tubular degeneration and interstitial fibrosis, and has been implicated in other endemic nephropathies such as Balkan endemic nephropathy [18],[19]. Urinary OTA and other mycotoxins can be detected in residents of MeN‑endemic communities [20], and experimental data suggest that coexposure to OTA and fumonisins (common contaminants of maize) may have additive or synergistic nephrotoxic effects [21], although such interactions have not been definitively demonstrated in MeN patients. Agrochemicals, including certain herbicides (e.g., paraquat, glyphosate) and insecticides, can induce oxidative stress and mitochondrial dysfunction in renal cells and have been proposed as environmental cofactors in MeN, though human data are heterogeneous [10]. Glyphosate, widely used in sugarcane production, is a strong chelator of divalent metals and has been linked to CKD in some epidemiologic and mechanistic reviews, raising the hypothesis that glyphosate–metal complexes could enhance nephrotoxicity, but causal evidence remains limited [22]. Heavy metals (arsenic, cadmium, lead, nickel) and silica from soil dust or water further implicate geogenic sources [7]. Arsenic, which is above WHO limits in some wells, causes tubular injury, while silica may trigger immune responses.

## 5. The gut–kidney axis and immune dysregulation

Recent insights emphasize gut–kidney interactions. Chronic malnutrition and recurrent enteric infections, prevalent in poverty-stricken areas, impair intestinal barrier function, leading to a “leaky gut”. Emerging data indicate MeN patients may exhibit elevated markers of gut permeability and altered microbiota composition, with immunoglobulin A (IgA) deposits in some biopsies suggesting a possible immune-mediated mechanism triggered by gut-derived antigens [23]. This permits microbial translocation, elevating circulating endotoxins and proinflammatory cytokines (e.g., interleukin (IL)-6, tumor necrosis factor alpha (TNF-α)). MeN biopsies show IgA deposits, suggesting antigen-driven immune complex formation. By analogy with other forms of CKD, gut microbiome dysbiosis—characterized by a loss of butyrate‑producing bacteria and increased production of gut‑derived uremic toxins—has been implicated in renal inflammation and progression [24],[25]. Metabolomic studies in CKD demonstrate altered energy and amino acid pathways and accumulation of gut‑derived uremic solutes, which may also be relevant to MeN's pathophysiology, although direct data in MeN remain limited [26]. Therefore, dedicated microbiomics/metabolomics studies in MeN are urgently needed.

## 6. Genetic and epigenetic modifiers

A recent genome-wide association study highlights a predisposition linked to Native American ancestry, with each 1% increase in ancestry associated with a 4.1% rise in the odds of MeN. Protective intronic variants in *OPCML* reduce the risk and may enhance urine osmolality and dehydration resistance. Variants in *NOS3* (*rs2070744*) may reduce nitric oxide production, contributing to endothelial dysfunction, impaired renal perfusion, and susceptibility to heat-induced ischemic injury [27]. Polymorphisms in the *APOE* gene (rs429358) have been associated with hyperuricemia, promoting oxidative stress, mesangial inflammation, and progressive tubulointerstitial damage. Epigenetic alterations involving *AMPH, SLC29A3*, and imprinted loci (DIO3, RTL1, DLK1) further implicate regulatory pathways influencing renal fibrosis and inflammatory signaling [28]. These findings suggest that the genetic background may amplify vulnerability to environmental and occupational stressors rather than acting as a primary cause.

**Table 1.** Proposed mechanisms associated with renal injury in MeN.

Category	Specific factor	Proposed mechanism of renal injury	Key supporting evidence	Potential synergies/interactions
Occupational heat stress	Recurrent dehydration and heat exposure during strenuous labor	Repeated subclinical AKI from volume depletion, hyperthermia, and rhabdomyolysis-like insults; leads to cumulative tubular damage, oxidative stress, and interstitial fibrosis	Cross-shift eGFR declines in sugarcane workers; seasonal AKI spikes; animal models of heat + dehydration showing fibrosis	Amplifies toxicity of co-exposures (e.g., lowers the threshold for toxin-induced damage); worsened by inadequate rest/hydration
Water contamination	Endotoxins (LPS) from Gram-negative bacteria in untreated shallow wells/rivers	Chronic low-grade activation of TLR4 on renal tubular cells → inflammation, NF-κB signaling, and progressive fibrosis	Elevated urinary LPS biomarkers in workers; rodent models showing LPS exacerbates AKI [3]; contaminated water sources in hotspots	Synergistic with heat stress (exercise + LPS worsens AKI); contributes to systemic inflammation via the gut–kidney axis
Dietary mycotoxins	OTA, fumonisins from moldy maize/beans in humid storage	Direct tubular toxicity → oxidative stress, mitochondrial dysfunction, lysosomal abnormalities, DNA damage, and apoptosis	OTA metabolites detected in the urine of affected workers; biomonitoring studies in Nicaragua/El Salvador	Co-exposure with agrochemicals enhances oxidative injury; heat/malnutrition may impair detoxification
Agrochemicals	Glyphosate, paraquat, other herbicides/pesticides	Oxidative stress, mitochondrial inhibition, disruption of cellular metabolism; possible metal chelation increasing toxicity	Epidemiological links in agricultural cohorts; experimental data on glyphosate's nephrotoxicity; high usage in sugarcane regions	Additive with mycotoxins and heavy metals; heat stress increases absorption/uptake
Heavy metals and geogenic toxins	Arsenic, cadmium, nickel, and lead from water/soil	Tubular necrosis, oxidative damage, interference with enzymatic function	Elevated urinary arsenic/cadmium in some cohorts; geogenic contamination in Pacific lowlands	Chelates with agrochemicals; chronic low-dose exposure synergizes with other oxidants
Inhalational exposure	Silica dust (agriculture, brickmaking)	Immune activation, inflammasome triggering, possible direct tubular injury	Reported in brickmakers and miners with MeN; histopathological similarities to silicosis-related kidney disease	Combines with heat and endotoxin exposure in dusty fields
Gut–kidney axis disruption	Leaky gut from malnutrition, recurrent infections, dysbiosis	Increased intestinal permeability → microbial translocation, elevated circulating endotoxins/cytokines → systemic and renal inflammation; uremic toxin retention	Elevated gut permeability markers; altered microbiota and metabolomes in MeN patients; IgA deposits in biopsies	Heat/dehydration worsens gut barrier; malnutrition common in affected communities amplifies the effect
Immune dysregulation	Chronic low-grade inflammation, possible antigen-driven response	Immune complex deposition, cytokine storm, amplification of tubular injury	IgA nephropathy-like features; elevated inflammatory markers in cohorts	Triggered by translocated gut antigens or environmental toxins; positive feedback with heat stress
Genetic susceptibility	Native American ancestry; variants in *OPCML, NOS3*, and *APOE*	Reduced NO bioavailability → endothelial dysfunction; hyperuricemia → OS and mesangial inflammation; impaired osmotic regulation → enhanced susceptibility to dehydration-induced AKI; amplification of TIF pathways	Genome-wide association studies: ancestry–risk correlation analyses; epigenetic profiling	Increases vulnerability to heat stress, dehydration, and environmental toxins

## 7. Pathophysiology: Cellular and morphological insights

Histologically, MeN features chronic tubulo-interstitial nephritis with fibrosis, glomerular sclerosis, and vascular changes, but minimal proteinuria, which distinguishes it from diabetic nephropathy. Animal models confirm that repeated LPS exposure can induce AKI and chronic renal injury [29]. Additionally, in hot, humid environments, poorly stored crops like maize and beans are prone to contamination with nephrotoxic fungal metabolites, including OTA and fumonisins, and exposure to agrochemicals like glyphosate may exacerbate oxidative stress. Biomonitoring studies in Nicaragua have detected OTA metabolites in urine samples, suggesting real-world relevance [20]. Morphological studies using electron microscopy have also revealed lysosomal abnormalities and mitochondrial dysfunction in tubular epithelial cells [30], suggesting that impaired cellular metabolism and oxidative stress may represent a convergent pathway through which multiple toxins act. While controversy remains, these findings support a multifactorial model in which environmental, toxic, immunologic, and occupational stressors jointly drive MeN's pathogenesis (Table 1).

## 8. A public health crisis in slow motion

The consequences of MeN extend beyond the clinic, creating a public health crisis in slow motion. Families lose providers, communities lose labor forces, and local health systems—already under-resourced—are overwhelmed. In many areas, dialysis is unavailable, and even basic diagnostics like creatinine testing are rare [22],[31]. Without early detection or effective therapy, MeN is almost uniformly fatal. Clinicians should consider occupational history in CKD evaluations to improve recognition.

Efforts to address MeN have encountered political and economic challenges. Some industry stakeholders have questioned the connection between MeN and occupational exposure, slowing progress toward reform [32]. However, pilot interventions, such as protocols involving hydration, rest, and shade and electrolyte supplementation, have shown promise in reducing the decline in kidney function among sugarcane workers [33],[34]. Regional efforts, including the Consortium on the Epidemic of Nephropathy in Central America and Mexico and the Pan American Health Organization's Resolution CD52.R8, have contributed to coordinated responses. Despite these, progress remains limited, and international involvement has been minimal [35].

As global temperatures rise and millions of laborers continue to work in hazardous outdoor conditions, the risk of heat- and toxin-induced CKD is expected to spread beyond Mesoamerica. Similar patterns are already suspected among agricultural workers in regions such as California and Florida in the southern United States [36],[37].

To strengthen the global response to MeN, the international health community may wish to consider several priority actions. These include supporting further research into the condition's multifactorial causes through environmental sampling, biomarker development, and multi-omics approaches; strengthening occupational protections by ensuring access to clean water, adequate rest breaks, and shade for workers in high-temperature settings; promoting community-level screening programs integrated with primary care services to facilitate early detection of kidney damage; and addressing systemic barriers so that the diagnosis and treatment of CKD become accessible in low-income and rural areas.

MeN underscores the importance of recognizing CKD not only as a condition associated with lifestyle or aging, but also as an occupational and environmental health problem closely linked to poverty and likely with climate change. The loss of tens of thousands of young workers in their most productive years represents a humanitarian emergency that extends beyond local or regional concern. As the climate continues to warm and labor conditions remain unequal, the insights gained from MeN are becoming increasingly relevant worldwide. Addressing this epidemic effectively requires sustained global cooperation, scientific focus, and a broader re-examination of public health strategies in the context of climate change and social inequity.

## Use of AI tools declaration

The authors declare they have not used artificial intelligence (AI) tools in the creation of this article.
